# Ocean feedback to pulses of the Madden–Julian Oscillation in the equatorial Indian Ocean

**DOI:** 10.1038/ncomms13203

**Published:** 2016-10-19

**Authors:** James N. Moum, Kandaga Pujiana, Ren-Chieh Lien, William D. Smyth

**Affiliations:** 1 College of Earth, Ocean and Atmospheric Sciences, Oregon State University, Corvallis, Oregon 97331-5503, USA; 2 Faculty of Earth Sciences and Technology, Bandung Institute of Technology, Bandung 40116, Indonesia; 3 Applied Physics Laboratory, University of Washington, Seattle, Washington 98105, USA

## Abstract

Dynamical understanding of the Madden–Julian Oscillation (MJO) has been elusive, and predictive capabilities therefore limited. New measurements of the ocean's response to the intense surface winds and cooling by two successive MJO pulses, separated by several weeks, show persistent ocean currents and subsurface mixing after pulse passage, thereby reducing ocean heat energy available for later pulses by an amount significantly greater than via atmospheric surface cooling alone. This suggests that thermal mixing in the upper ocean from a particular pulse might affect the amplitude of the following pulse. Here we test this hypothesis by comparing 18 pulse pairs, each separated by <55 days, measured over a 33-year period. We find a significant tendency for weak (strong) pulses, associated with low (high) cooling rates, to be followed by stronger (weaker) pulses. We therefore propose that the ocean introduces a memory effect into the MJO, whereby each event is governed in part by the previous event.

## Abstract (web summary)

The Madden-Julian Oscillation (MJO) describes an eastward propagating pulse of tropical convection. Here, using short-term field measurements, Moum *et al*. illustrate an MJO memory effect: strong pulses drive enhanced ocean heat loss, weakening subsequent pulses, with implications for MJO prediction.

## Graphical abstract

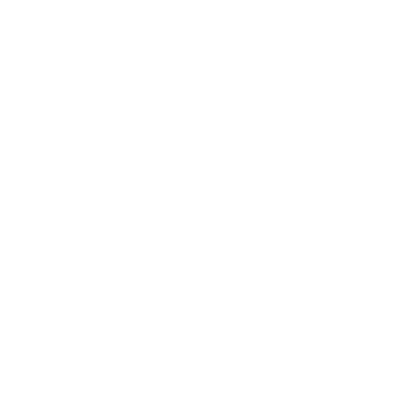

The Madden–Julian Oscillation (MJO) is a tropical intraseasonal oscillation that forms in the Indian Ocean and whose dominant surface signature in the equatorial Indian and western Pacific Oceans is a pulse of intense winds and heavy precipitation coupled with deep atmospheric convection lasting 2–3 days at a given location, covering an area of 50,000 km^2^ and recurring at intervals of 30–90 days^1^. Its signal propagates around the globe in the equatorial band, where it influences weather phenomena as disparate as Atlantic hurricanes and the Pineapple Express along the west coast of North America^2^. Here, we use the term ‘pulse' to represent the westerly wind burst at the sea surface, beginning at the time when the surface wind stress exceeds a threshold value and ending when the stress subsequently falls below this value.

The effect of sea surface temperature (SST) on some forms of weather phenomena is well established. For example, hurricanes derive most of their energy from heat stored in the upper ocean^3^. The significance of diurnal warm layers on the intensity of atmospheric convection has been shown through simulations^4^ and observations^5^. It has been suggested that, in March 2015, anomalously warm SST in the western Pacific provided the heat energy for MJO amplification to record amplitudes^6^. However, neither the role of air–sea fluxes^7^,^8^ nor that of internal ocean processes^9^,^10^ in MJO development is clear at present.

By virtue of its high heat capacity (4 × that of air) and density (10^3^ × air), the ocean responds slowly to energetic, rapidly moving and evolving atmospheric disturbances like the MJO. These same factors also mean that the oceanic response continues after the atmospheric disturbance has passed. They raise the issue that there may be an effect of this slowly evolving response on subsequent atmospheric disturbances.

Here, we link insight gained from recent short-term field measurements with a statistical analysis of the existing longer-term record to show an unanticipated dependence of MJO intensity on SST. Dynamics of the Madden–Julian Oscillation (DYNAMO)^11^,^12^ was a large-scale air–sea interaction experiment in the central equatorial Indian Ocean in boreal autumn of 2011 that included local and coincident measurements of surface fluxes and subsurface currents, stratification and mixing. Early results from shipboard measurements made during this experiment showed the persistence of the subsurface disturbance following passage of the MJO in the atmosphere^13^. Here we include longer-term moored records, also part of DYNAMO, that demonstrate the full subsurface response to two consecutive MJO pulses, a strong pulse following a weak pulse. Since it was at least possible that the greater upper ocean heat content (HC) remaining after the weak pulse might have contributed to the stronger following pulse, this anecdotal result prompted us to pose a hypothesis that stronger (weaker) pulses always follow weak (strong) pulses. To address this hypothesis we exploited a set of reanalysis data products that begins in 1980 and includes sufficient information to identify MJO pulses, and to quantify their intensity as well as pre- and post-pulse SST. These data revealed 84 MJO pulses between 1980 and 2013. Of these, 18 pulse pairs separated by <55 days were identified. These pulse pairs showed a significant correlation between SST cooling produced by the initial pulse and the intensity of the following pulse. The change in SST also correlated negatively with the intensity of each MJO pulse. Taken together, these suggest negative feedback between successive MJO pulses, thus supporting our stated hypothesis.

## Results

### Details of upper ocean response to two successive MJO pulses

Subsurface measurements during and following pulses of the MJO in the Indian Ocean suggest that the ocean's response may influence subsequent pulses. These measurements have led to distinctions in both form and intensity between observed pulses^12^,^13^,^14^. Two pulses in October and November 2011 were particularly well-sampled and helped in illustrating differences between relatively weak and relatively strong pulses. We next assess the generality of these patterns using historical data.

**Figure 1:**
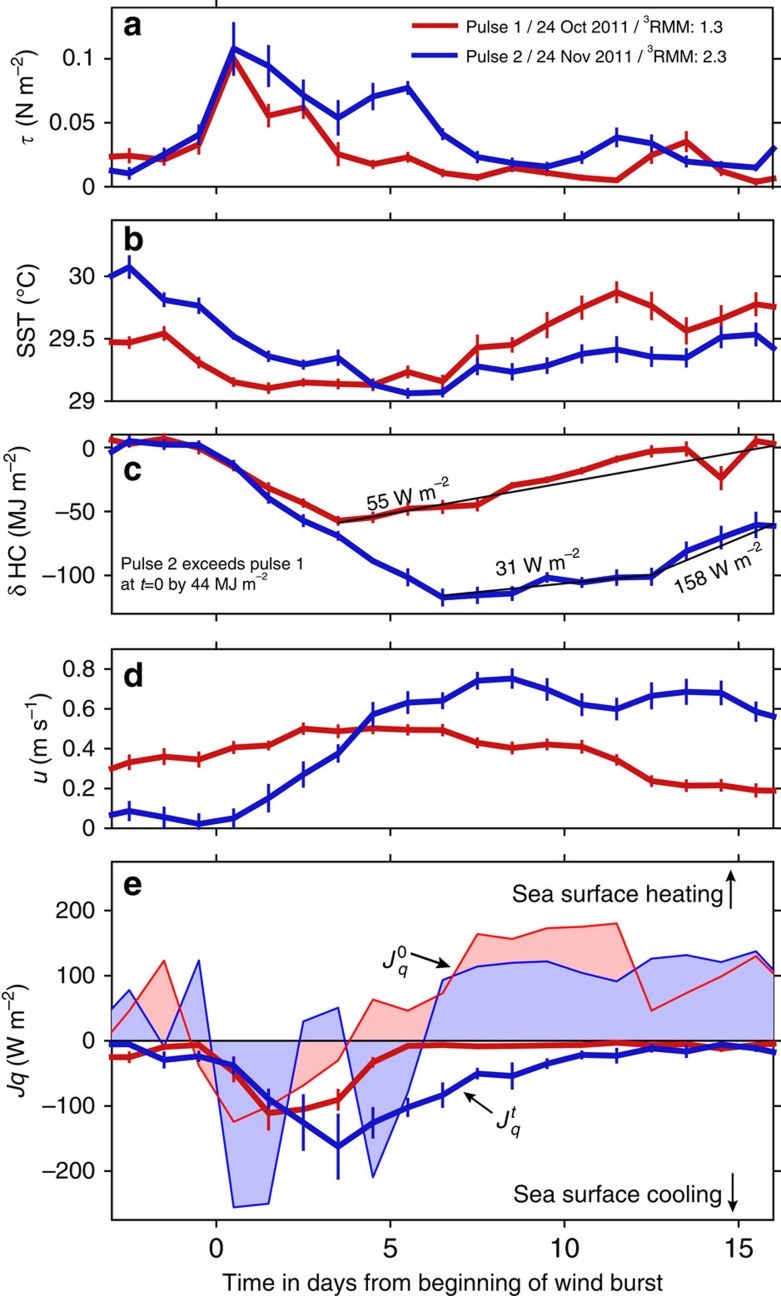
Ocean response to successive Madden–Julian Oscillation (MJO) pulses. The first, relatively weak (red; ^3^RMM=1.3) and second strong (blue; ^3^RMM=2.3) pulses were sensed at oceanographic moorings at 0°,78° E; 0°,80° E and 0°, 90° E during the DYNAMO experiment in boreal fall 2011. The time base (in days) is relative to the beginning of the principal wind burst of each pulse at each location with the arrival date at 0, 80° E noted in the legend. The timing at each location has been lagged at the nominal MJO propagation speed of 5 m s^−1^ to match and then variables shown averaged in normalized time. (**a**) Surface wind stress, *τ*. (**b**) Sea surface temperature, SST. (**c**) Heat content anomaly, upper 40 m. Heat content, 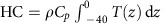, where *ρ* is the density and *C*_*p*_ the specific heat of seawater, *T*(*z*) is the depth-dependent temperature profile from temperature sensors on the moorings and the integral is computed over the upper 40 m of the water column. The anomaly is referenced to the average value of HC over the three days before day 0. Before pulse 2, HC was larger by 44 MJ m^−2^. The thin, annotated black lines represent heating rates of the upper 40 m following cessation of the wind bursts. (**d**) Zonal current velocity, *u*, (eastward>0) averaged over the ocean's upper 100 m. (**e**) Daily-averaged heat flux. 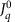 is the net surface heat flux and 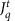 is the subsurface turbulent heat flux. 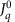 is >0 when dominated by solar heating and the ocean surface is heated. 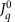 is <0 when dominated by latent cooling. 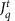 represents downgradient heat transfer by turbulence in the ocean and is almost always <0, cooling the sea surface. Thick lines represent 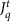, thin lines 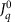. The shading emphasizes the sign of 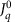. The vertical bars represent 95% bootstrap confidence limits.

A large-scale quantification of the intensity of each pulse is provided by the real-time multivariate MJO (RMM) index^15^. RMM provides an assessment based on outgoing longwave radiation (OLR) and winds at 200 and 850 hPa. RMM has both amplitude and phase, where phase roughly references geographic regions around Earth at the equator. The central equatorial Indian Ocean, the region of detailed measurements discussed here, roughly corresponds to phase 3 (ref. 16), and we refer to the value of RMM computed there as ^3^RMM. A value of ^3^RMM>1 is considered to be a significant event. The examples in Fig. 1 are characterized by ^3^RMM=1.3 (significant but relatively weak pulse) and ^3^RMM=2.3 (strong pulse), where these values represent three-day averages centred at the peak daily value.

The two pulses were sensed at three locations along the equator where detailed subsurface measurements were made (0°, 78° E; 0°, 80° E; 0°, 90° E) from oceanographic moorings instrumented with turbulence sensors^17^. An averaged oceanic response was derived by shifting the time series using the nominal MJO pulse propagation speed (5 m s^−1^) to reference time at each location to the arrival of the pulse. Time series of surface wind stress (*τ*), SST, zonal velocity (*u*), heat content anomaly (δHC), net heat flux at the sea surface (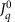) and subsurface turbulent heat flux (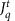) were averaged daily.

Significant distinctions are apparent between the relatively weak early MJO pulse and the later, stronger pulse (Fig. 1). During the stronger pulse surface forcing was greater and lasted longer, represented by large *τ* (Fig. 1a) and large negative 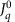 (Fig. 1e). Before pulse 2, SST was 0.5 K warmer than it was before pulse 1 (Fig. 1b), after which it cooled by 1.0 K, compared with a net cooling of <0.5 K for pulse 1. The high SST preceding the MJO pulse has been referred to as the positive intraseasonal SST anomaly^18^,^19^. Upper ocean HC (integrated over 0–40 m) before pulse 1 was smaller than it was before pulse 2 by 44 MJ m^−2^. Following pulse 1, cooling led to a minimum value δHC=−55 MJ m^−2^ 4 days after the wind burst when 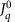 reversed sign to heating, and the upper ocean was heated at a rate of 55 W m^−2^, recovering the full heat lost by the pulse after 16 days (Fig. 1c). In contrast, seven days after pulse 2, the ocean had lost 117 MJ m^−2^ of heat energy. The initial recovery coincided with the sign reversal of 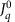, but was weaker (31 W m^−2^) until day 13, followed by rapid heating (158 W m^−2^). After 16 days, the HC was still smaller by 60 MJ m^−2^ than its initial value and indeed, smaller than the pulse 1 minimum. Note that the cooling of SST during the disturbed (cloudy) state before the WWB is not reflected in δHC. It is primarily due to a deeper and weaker diurnal warm layer.

The greater wind stress of pulse 2 accelerated an eastward jet in the upper 100 m of the ocean by 0.8 m s^−1^ over 8 days (Fig. 1d), causing a doubling of eastward mass transport^13^, a deepening of the jet and an enhancement of shear at its base. The combination of strong surface forcing and enhanced shear is presumably responsible for the increased and persistent subsurface turbulence, expressed here as 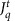 (Fig. 1e), the average turbulent heat flux^20^ over the upper 79 m (see ‘Methods' section). The subsurface cooling inferred from 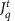 through down-gradient, Fickian-like diffusion (deeper waters are cooler) was large for at least an additional 7 days past the cessation of the wind burst. The slope change in δHC after pulse 2 coincides with diminished 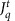.

Larger and extended cooling of the upper ocean during the passage of pulse 2 is consistent with larger and extended surface forcing. But after the passage of the wind burst, δHC and SST recovery rates were considerably less than following pulse 1, despite comparable net surface heating. Figure 1e suggests that cooling from below via subsurface mixing is the likely cause of this. The net result is that the stronger MJO pulse reduces upper ocean HC more than the weaker pulse does, and this cooling continues after the passage of the atmospheric disturbance. We next assess the generality of these patterns using historical data.

### Properties of MJO pulses 1980–2013

The differences in oceanic response to the varying intensity of the pulses is important if upper ocean HC is a significant contributor to MJO pulse intensity. This would be the case if the greater heat available following a weak pulse feeds back into a stronger following pulse. We test this idea using a longer record (1980–2013) of atmospheric and surface variables derived from reanalysis products of daily, gridded estimates of OLR (ref. 21), *τ*, SST and 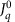 (ref. 22) in the tropics. Data were averaged over 3 days in time, and spatially over 6° of latitude and 6° of longitude centred at 0°, 80° E, roughly the centre of RMM phase 3. These data provide a broader spatial perspective than is obtained from the set of point measurements shown in Fig. 1. From these data, MJO pulse pairs were selected under the following criteria: we required that ^3^RMM>1, that pulses propagated eastward in OLR at 3–9 m s^−1^, that zonal winds at 850 hPa (National Centers for Environmental Prediction (NCEP) reanalysis) were westerly and that the pulse pair separation was <55 days. The resulting pulse durations ranged from 3 days (which is our averaging period, hence minimum length) to 14 days with mean and median of 6 days.

**Figure 2:**
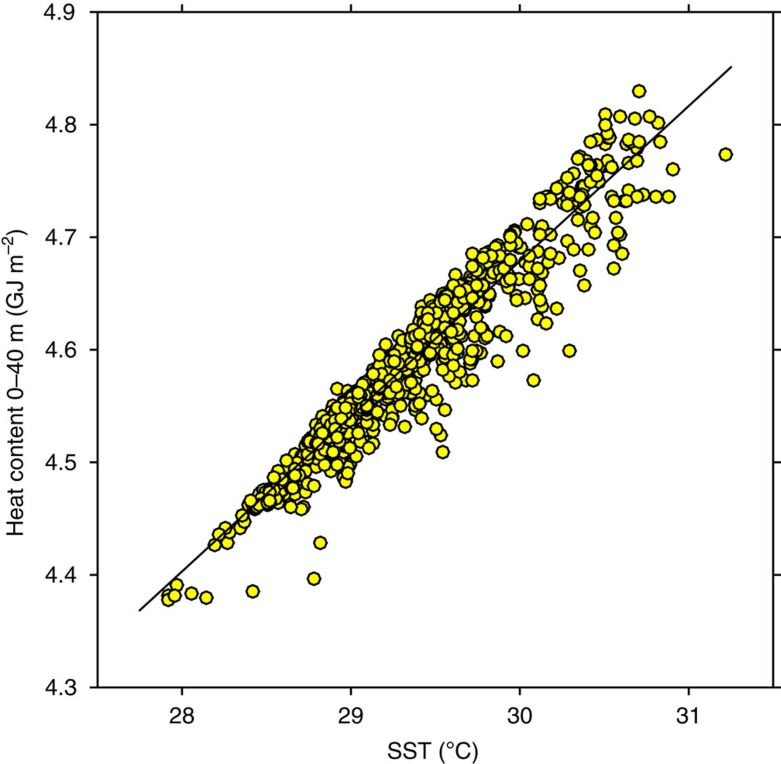
Heat content versus SST. Heat content (HC) derived from daily-averaged temperature profiles (integrated over the upper 40 m) and SST at the RAMA mooring at 0°, 80° E between late 2008 through 2012. The 905 data points are significantly correlated, *r*=0.95 [0.94, 0.96] where the values in [ ] represent 95% confidence limits. The linear regression indicated by the black line is HC=0.14 × SST+0.54.

In our analysis, we use SST as a proxy for HC. While it is SST that directly communicates heat from ocean to atmosphere, a thin layer cannot do so for very long. A more representative measure of the ocean's capacity to effect significant influence on the atmospheric boundary layer above is obtained by integrating over the upper ocean to determine its HC. To assess the correspondence of SST to HC, subsurface temperature and SST data from the Research Moored Array for African-Asian-Australian Monsoon Analysis and Prediction (RAMA) mooring at 0°, 80° E between late 2008 and through 2012 was averaged daily and HC computed over the upper 40 m. The comparison of HC and SST is shown in Fig. 2. The strong correlation between the two provides a rationale for using SST as a proxy for HC.

**Figure 3:**
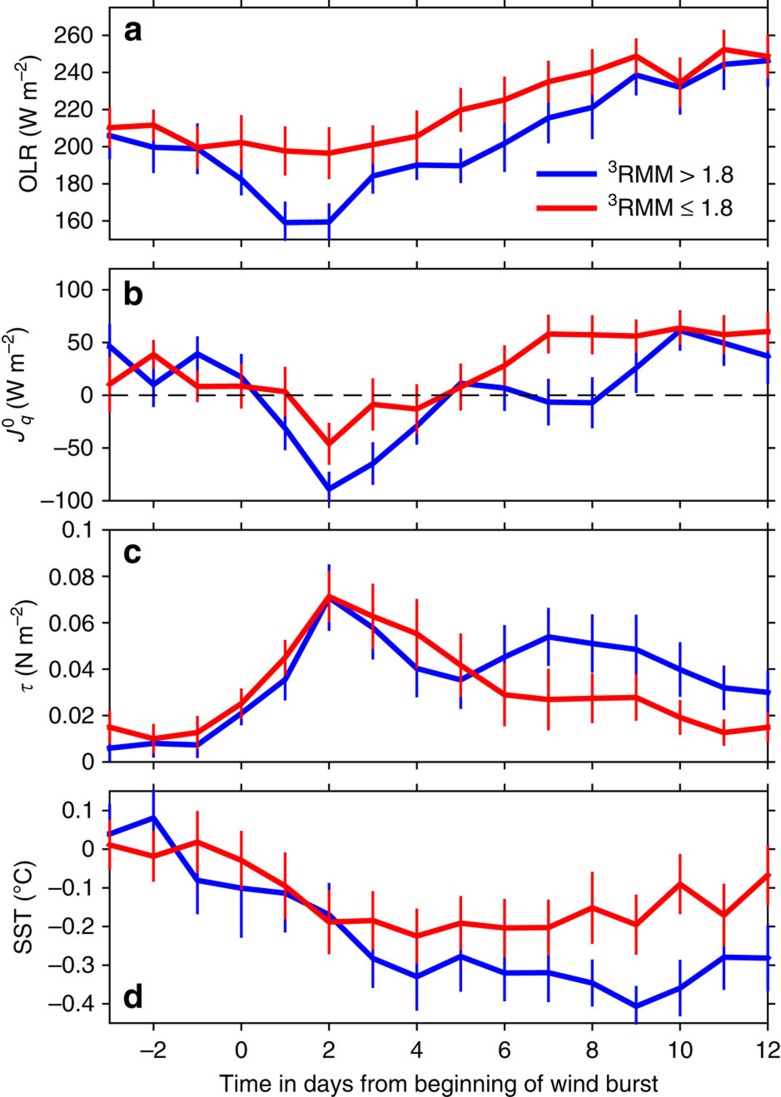
Composite averaged temporal evolution of paired pulses. The pairs are separated by<55 days, as identified in the text. Ensemble averages derived from 18 weak (1<^3^RMM<1.8; red) and 18 strong pulses (^3^RMM>1.8; blue). Time is referenced to the principal wind burst of each pulse. (**a**) Outgoing longwave radiation, OLR. (**b**) Net surface heat flux, 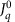. (**c**) Surface wind stress, *τ*. (**d**) Sea surface temperature, SST. The vertical bars represent 95% bootstrap confidence limits.

From the complete records starting in 1980, our selection criteria delivered 18 MJO pulse pairs. The beginning of each pulse was identified as the time at which westerly *τ* increased above 0.025 N m^−2^, accompanied by a sign change in 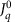 from surface heating to surface cooling. The end of each pulse coincides with *τ* decreasing below the same threshold value. The data series generated from reanalysis products were referenced to the beginning of the principal wind burst, so that resulting series are in terms of time since the beginning of the wind burst. They were then simply averaged into composite weak (^3^RMM≤1.8) and strong (^3^RMM>1.8) time series; their characteristics are depicted in Fig. 3. The stronger (blue) MJO pulses are characterized by larger negative OLR anomalies indicating cooler cloud tops, hence deeper convection (Fig. 3a), greater cooling of the sea surface (Fig. 3b) and larger and longer-lasting surface wind stress (Fig. 3c). SST cooling (Fig. 3d) begins before the principal wind burst (as in Fig. 1) presumably during the disturbed state preceding the active MJO. As in the DYNAMO pulses represented in Fig. 1, there is no reason to suspect that upper ocean HC decreases since 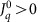 during this period. This early decrease in SST is likely due to suppression of the near-surface diurnal warm layer. Net SST cooling is, on average, 0.25 K for weak pulses, the minimum occurring 5 days after the wind burst begins and 0.5 K for strong pulses, with the minimum occurring 10 days after the beginning of the wind burst (*t*=0). Consequently, after 30 days, SST has fully recovered to its pre-pulse value after the weak pulses but is still 0.3 K smaller 30 days after the strong pulses.

A particular feature to note in Fig. 3c is the twin peaked structure in wind stress during strong MJO pulses. The peaks are separated by 5 days, as in the stronger, well-sampled DYNAMO pulse in Fig. 1. The signal in Fig. 1 has been associated with a pair of atmospheric Kelvin waves tracked in satellite precipitation records and embedded within the greater envelope of the propagating MJO pulse^13^. Is it possible that stronger MJO pulses are always associated with convectively coupled Kelvin waves in the central equatorial Indian Ocean?

### Stronger MJO pulses cause greater SST cooling

**Figure 4:**
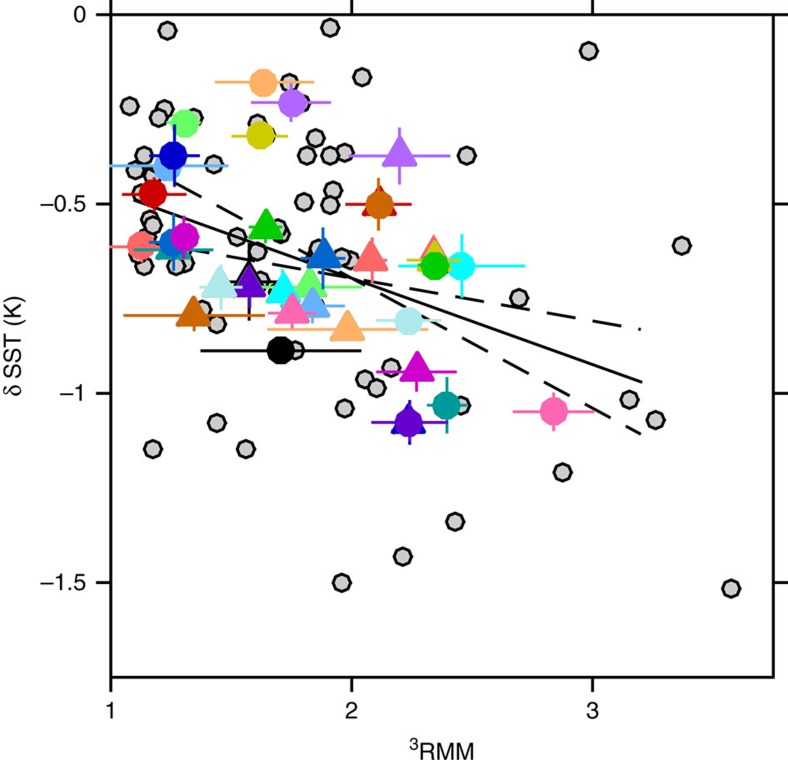
Net surface cooling of 84 MJO pulses relative to ^3^RMM. Data represent pulses identified using reanalysis data products between 1980 and 2013, as described in the text. Of these 84 pulses (grey circles), 18 pulse pairs separated by <55 days are plotted with matching colours, each colour representing a pair of pulses. The first of the pair are denoted by circles, the second by triangles. Confidence limits are defined in the Methods. The pulse pair data are significantly and negatively correlated with *r*=−0.49 [−0.71, −0.20], *P* value=0.0022. The solid black line represents the regression δSST=−0.23 × ^3^RMM−0.2. The dashed black lines represent 95% confidence limits on the slope of the regression. Correlations, slopes and *P* values are not substantially different between the 84 parent pulses and the 36 paired pulses.

For each individual pulse, the net sea surface cooling was determined as the SST difference between post-wind burst minima (for *t*<15 days) and the value at *t*=0. In Fig. 4, paired MJO pulses are colour coded, the first in the pair (pulse_*n*_) denoted by a circle and the second (pulse_*n*+1_) by a triangle. The stronger pulses (quantified by larger ^3^RMM, an index independent of SST) are associated with stronger SST cooling (δSST in Fig. 4). If SST is a reliable proxy of HC, then Fig. 4 indicates that, on average, pulses following strong initial pulses pass over an upper ocean with reduced HC compared with those following weak pulses.

### Negative feedback between pulse pairs

**Figure 5:**
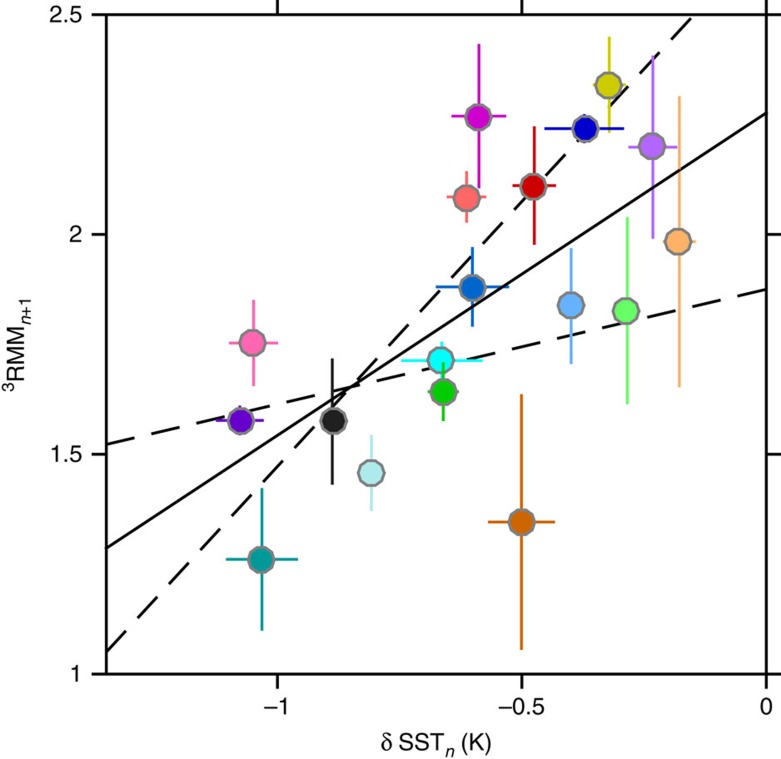
Negative feedback. Following pulse intensity (^3^RMM_*n*+1_) is plotted as a function of SST cooling from the initial pulse (δSST_*n*_). The colours identify each of the paired pulses in Fig. 4. Confidence limits are defined in the Methods. The data are significantly correlated with *r*=0.64 [0.24, 0.85], *P* value=0.0046. The solid black line represents the regression ^3^RMM_*n*+1_=0.73 × δSST_*n*_−2. The dashed black lines represent 95% confidence limits on the slope of the regression.

Apparently, the atmosphere responds to these changes in ocean HC, at least over relatively short time scales (<55 days). When we compare the intensities of following pulses (^3^RMM_*n*+1_) to SST changes caused by the corresponding initial pulses (δSST_*n*_), there is significant correlation (Fig. 5). This correlation progressively disappears when the time between pulses exceeds 55 days. The sense of the correlation in Fig. 5 is such that weak (strong) SST cooling is followed by a stronger (weaker) pulse. This is consistent with a scenario in which short-term changes in upper ocean HC feed back (negatively) to the intensity of MJO pulses, acting as a short-term governor on the system.

### Stronger MJO pulses generally follow larger pre-pulse SST

**Figure 6:**
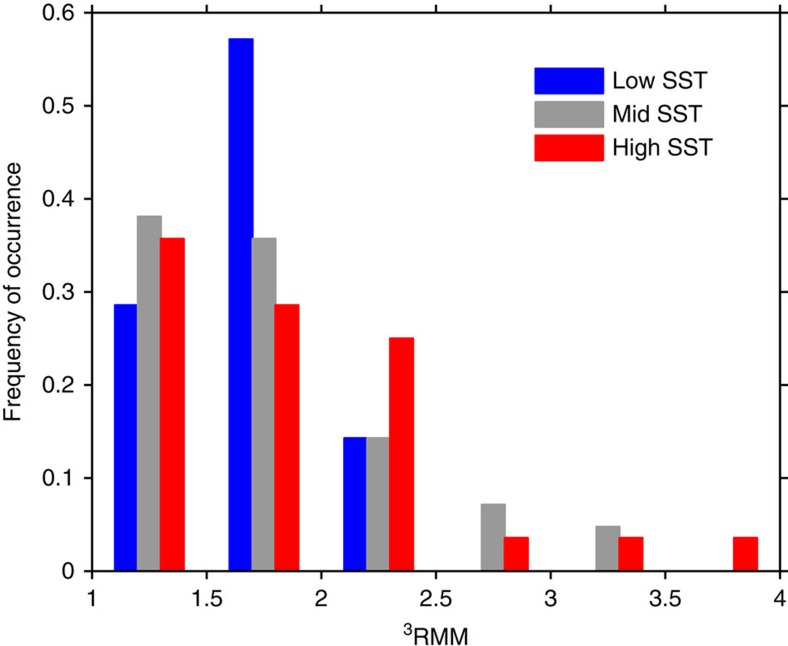
Stronger MJO pulses are generally associated with higher pre-pulse SST. Frequency of occurrence of 84 MJO pulse ^3^RMM values with the following ranges of pre-existing SST: low SST (<29 °C; blue), mid-range SST (29–30 °C; grey) and high SST (>30 °C; red).

The correlation shown in Fig. 5 is also consistent with a broader potential association of high SST and high ^3^RMM (Fig. 6). The occurrence and intensity of tropical cyclones (TCs, including hurricanes and typhoons) is dynamically associated with warm SST through its effect on the moisture content of the atmospheric boundary layer. However, showing this statistically (important for projecting occurrence frequencies and intensities in a changing climate) has not been straightforward. Tropical SST has been shown to be correlated with the maximum cubed surface wind speed within hurricanes, a direct measure of the power in individual TCs (ref. 23). A strong relationship also exists between the frequency of TCs with SST (ref. 24), however indirect the SST/TC number relationship. These studies benefited from relatively long records (>80 years) and direct measurements of cyclone winds and occurrences.

By comparison, our record of ^3^RMM is short and measurements are less direct. However, a relatively unsophisticated analysis using ^3^RMM of the 84 MJO pulses identified in our record compared with SST averaged locally at 0°, 80° E is suggestive. We sorted the 84 pulses into three ranges of pre-existing SST and then counted the number of pulses in six ranges of ^3^RMM (Fig. 6). This shows a progressively greater propensity for large values of ^3^RMM to occur with progressively larger SST.

It is possible that ^3^RMM is not the best metric for this analysis. ^3^RMM is not a direct measure of energy while SST, as a proxy for HC, is. A more insightful analysis might potentially use SST over a broader equatorial expanse of the Indian Ocean to include the origins of the MJO pulses.

## Discussion

For the two MJO pulses we have been able to observe in detail the total heat extracted from the upper ocean is lost in roughly equal parts to the atmosphere via 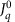 and to the deeper stratified ocean via 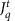 (Fig. 1). Through the first MJO, 19 MJ m^−2^ was lost to surface fluxes over the first 3 days of the wind burst and 24 MJ m^−2^ lost to subsurface fluxes over 5 days. In the second MJO pulse, 69 MJ m^−2^ of heat energy was lost to surface fluxes over 2 two-day periods associated with each individual wind burst and 85 MJ m^−2^ to subsurface fluxes over 12 days. The extended cooling from below is due to turbulence generated via instabilities by the persistent shear at the base of the Yoshida–Wyrtki Jet that is excited by the wind bursts. Roughly 25% greater cooling is attributed to subsurface fluxes compared with surface fluxes.

Contrast this to two extreme cases. In the open ocean in moderate winds and away from strong currents, nighttime convection is principally due to cooling of the ocean from above by the atmosphere. A little more than 10% of the cooling of the mixed layer is effected by mixing of cooler waters across the mixed layer base through penetrative convection^25^. At the other extreme, TCs extract their energy from the ocean, which contributes several thousands of W m^−2^ to cooling the upper ocean during cyclone passage^26^. In this case subsurface fluxes are 5–10 × greater than surface fluxes. These two extremes can be characterized by 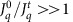 (nighttime convection) and 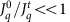 (TC). The MJO pulses observed here represent 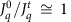.

In the case of the TCs, the redistribution of heat between near-surface and deeper ocean layers is so great that it has been suggested (via modelling) that the resultant deep warm anomalies, or deep mixed layers persist long enough to act as positive feedback to subsequent TCs^27^. That is, the sense of the correlation is opposite to that shown in Fig. 5. The deeper mixed layers induce smaller surface cooling as subsequent TCs pass, thus promoting stronger cyclone intensity. That the opposite correlation is found in Fig. 5 for MJO feedback must be due to the far less extreme values of 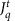 during and following the passage of MJO pulses.

The correlation in Fig. 5 suggests a significant influence of MJO pulses on those immediately following, indicating that the MJO leaves a memory of its passage behind as it propagates around the globe. The 55-day time scale is consistent with the relatively slow response time of the ocean, suggesting that the memory effect reflects heat storage in the upper ocean.

If the change in upper ocean HC effected by a pulse influences the following pulse, and the ocean contributes as much to reducing ocean HC via subsurface fluxes as does the atmosphere via surface fluxes, then there ought to be a general improvement in forecast skill by proper inclusion of subsurface mixing. The influence of the ocean on the atmosphere is via surface fluxes through the air–sea interface, which depend in part on SST. We have shown that subsurface fluxes contribute significantly to SST. Therefore, surface fluxes and internal ocean processes must both contribute to MJO intensity.

## Methods

### Identification of the pulse pairs in Figs 4 and 5

The Wheeler–Hendon computation of ^3^RMM uses OLR and winds at 200 and 850 hPa to make a fairly good predictor of MJO-like activity in the equatorial Indian Ocean. However, it does not guarantee eastward propagation of the OLR anomaly nor does it guarantee surface westerlies. These are further factors we have flagged for in our definition of MJO pulses. We select significant ^3^RMM values occurring between September and May and also require that: eastward propagation of the OLR anomaly with a phase speed of 3–9 m s^−1^, winds at 200 hPa and 850 hPa are out of phase and winds are westerly at 850 hPa.

### Confidence limits in Figs 4 and 5

For each MJO pulse, δSST was computed as the difference in SST at its minimum (within 15 days following pulse onset) and the value at pulse onset. Confidence limits were estimated as the sum of the confidence limits of the minimum and maximum values of SST over the averaging domain (3 × 3 degree box centred at 0° N, 82.5° E). In turn, these are 1.96*σ*/√*n*, where *σ* is the s.d., *n* the number of data points (36 one-degree resolution data points per day) and the factor 1.96 represents 95% confidence for a normal distribution.

The same approach was used to estimate confidence limits for ^3^RMM. We determined *σ* for ^3^RMM values within the averaging time window of 3 days.

### Definition of 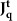


The vertical (more strictly, diapycnal) flux of heat by turbulence is represented by Fickian diffusion, enhanced by a turbulence diffusion coefficient, 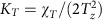 (ref. 28). The temperature-variance dissipation rate (*χ*_*T*_) is computed by scaling temperature-gradient spectrum measured by fast thermistors on small autonomous instruments (χpods^20^) attached to oceanographic moorings and packaged with inertial navigation units to quantify the component of flow speed past the sensor that is due to wave-induced motions of the surface float^29^. The other component of the flow speed is the ocean current speed, measured separately by velocity sensors on the mooring. The vertical temperature gradient, *T*_*z*_, is defined locally by the vertical motion of the sensor through the water or, on a larger scale, by additional temperature sensors on the mooring. The depth-dependent vertical heat flux caused by turbulence is 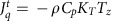, where *ρ* is the density of seawater and *C*_*p*_ its heat capacity. For this experiment, χpods were distributed differently in depth for each of the three moorings and we simply averaged 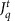 over all depths (in Table 1) at each mooring to provide relative estimates of subsurface heat flux between the weak and strong pulses in Fig. 1.

### External databases

^3^RMM index: daily data, http://www.bom.gov.au/climate/mjo/graphics/3RMM.74toRealtime.txt

OLR: daily data with a spatial resolution of 1/4 degree. Source: http://www.esrl.noaa.gov/psd/data/gridded/data.interp_OLR.html (ref. 21)

Zonal wind: daily NCEP reanalysis data. data: http://www.esrl.noaa.gov/psd/data/gridded/data.ncep.reanalysis.html

Wind stress and surface heat flux: daily TropFlux data with a spatial resolution of 1 degree. http://www.incois.gov.in/tropflux/ (ref. 22)

### Data availability

The data that support the findings of this study are available from the corresponding author upon request.

## Additional information

**How to cite this article:** Moum, J. N. *et al*. Ocean feedback to pulses of the Madden–Julian oscillation in the equatorial Indian ocean. *Nat. Commun.* **7,** 13203 doi: 10.1038/ncomms13203 (2016).

## Figures and Tables

**Table 1:** Moored data used in Fig. 1.

**Location**	**Temperature/HC**	**Zonal velocity/*****u***	**χpods/*****J***_***q***_^***t***^
0°, 78° E	1,5,10,15,20,30,40 m	1,5,10,15,20,30,40 m	24,37,43,59,79 m
0°, 80° E	1,5,10,13,20,40 m	10,35,40 m	24,34,44,59,75 m
0°, 90° E	1,10,13,20,40 m	10,35,40 m	14,21,58,62 m

Moorings at the equator and 80° E, 90° E are part of the long term RAMA array of equatorial Indian Ocean moorings^30^. The mooring at 0**°**, 78° E was deployed specifically for the DYNAMO experiment by the Applied Physics Lab at University of Washington. All three moorings were equipped with meteorological sensors from which the wind stress and net surface heat flux were derived from bulk formulae^31^. This table shows depths of velocity, temperature and χpod measurements on each mooring. HC; heat content.
